# Real-World Performance of the MiniMed™ 780G System: First Report of Outcomes from 4120 Users

**DOI:** 10.1089/dia.2021.0203

**Published:** 2022-01-31

**Authors:** Julien Da Silva, Giuseppe Lepore, Tadej Battelino, Arcelia Arrieta, Javier Castañeda, Benyamin Grossman, John Shin, Ohad Cohen

**Affiliations:** ^1^ Medtronic International Trading Sàrl, Tolochenaz, Switzerland.; ^2^ Unit of Endocrine Diseases and Diabetology, ASST Papa Giovanni XXIII, Bergamo, Italy.; ^3^ University Children's Hospital, University Medical Centre Ljubljana, and Faculty of Medicine, University of Ljubljana, Ljubljana, Slovenia.; ^4^ Medtronic Bakken Research Center, Maastricht, The Netherlands.; ^5^ Medtronic, Northridge, California, USA.

**Keywords:** Real-world evidence, Automated insulin delivery, Time-in-range, Hypoglycemia, Hyperglycemia, Diabetes

## Abstract

***Background:*** The MiniMed™ 780G system includes an advanced hybrid closed loop (AHCL) algorithm that provides both automated basal and correction bolus insulin delivery. The preliminary performance of the system in real-world settings was evaluated.

***Methods:*** Data uploaded from August 2020 to March 2021 by individuals living in Belgium, Finland, Italy, the Netherlands, Qatar, South Africa, Sweden, Switzerland, and the United Kingdom were aggregated and retrospectively analyzed to determine the mean glucose management indicator (GMI), percentage of time spent within (TIR), below (TBR), and above (TAR) glycemic ranges, system use, and insulin consumption in users having ≥10 days of sensor glucose (SG) data after initiating AHCL. The impact of initiating AHCL was evaluated in a subgroup of users also having ≥10 days of SG data, before AHCL initiation.

***Results:*** Users (*N* = 4120) were observed for a mean of 54 ± 32 days. During this time, they spent a mean of 94.1% ± 11.4% of the time in AHCL and achieved a mean GMI of 6.8% ± 0.3%, TIR of 76.2% ± 9.1%, TBR <70 of 2.5% ± 2.1%, and TAR >180 of 21.3% ± 9.4%, after initiating AHCL. There were 77.3% and 79.0% of users who achieved a TIR >70% and a GMI of <7.0%, respectively. Users for whom comparison with pre-AHCL was possible (*N* = 812) reduced their GMI by 0.4% ± 0.4% (*P* = 0.005) and increased their TIR by 12.1% ± 10.5% (*P* < 0.0001), post-AHCL initiation. More users achieved the glycemic treatment goals of GMI <7.0% (37.6% vs. 75.2%, *P* < 0.0001) and TIR >70% (34.6% vs. 74.9%, *P* < 0.0001) when compared with pre-AHCL initiation.

***Conclusion:*** Most MiniMed 780G system users achieved TIR >70% and GMI <7%, while minimizing hypoglycemia, in a real-world condition.

## Introduction

The paradigm shift in the treatment of type 1 diabetes mellitus (T1DM) from regimens based on extrapolation of historical data to algorithm-controlled insulin delivery systems based on real-time continuous glucose monitoring (CGM) has changed the clinical landscape by providing new therapeutic targets, as well as a leap in the proportion of people with T1DM safely achieving these goals. The new parameters are born from the growing use of CGM measurements of time spent in different ranges with consensus on definitions of ranges and current goals.^1^ For people with T1DM, except during pregnancy, the goals are to spend >70% of the time between the range of 70–180 mg/dL, <4% below 70 mg/dL, <1% below 54 mg/dL, and <25% above 180 mg/dL. These recommended durations of time spent across glucose ranges are likely appreciated by people with T1DM as they provide a clinically meaningful report on time spent in the extremes of hypoglycemia and hyperglycemia.^2^ Although measures of central tendency of glucose values (i.e., mean glucose and HbA1c) provide well-established predictors of future diabetes-related complications, measurement of time spent in ranges provides patient-centric information for correcting glycemic control, as well as a means by which to compare various therapeutic interventions. For example, a recent prospective study demonstrated the combined and independent contribution of improved time-in-range (TIR) and HbA1c in reducing microalbuminuria.^3^

The MiniMed 670G system (Medtronic, Northridge, CA, USA), the first hybrid closed-loop system approved for use, demonstrated the ability for users to reach current glycemic targets in clinical studies^4^ and, more recently, in real-world evidence (RWE) where 14,899 users from 13 countries achieved a mean glucose management indicator (GMI) of 7.0% ± 0.4%, TIR of 72.0% ± 9.7%, and time below range (TBR) <70 of 2.4% ± 2.1%.^5^ In addition, MiniMed 670G system use resulted in 58.9% and 61.5% of users achieving the recommended target goals for GMI <7% and TIR >70%, respectively. This surpasses the overall glycemic control reported in national and international registries where the majority of patients are not within the glycemic goals.^6–8^ Improved glycemic control demonstrated by automated insulin delivery (AID) systems^9,10^ has become the focus of how diabetes management will progress^9^ with the ultimate goal of tight glycemic control with lowered burden on people with T1DM, their caregivers, and health care providers.

The MiniMed 780G system contains an advanced hybrid closed-loop (AHCL) algorithm that incorporates innovation derived from the MiniMed 670G system. The technology includes every 5-min automatic basal insulin delivery, adjustable targets of 100 (5.5), 110 (6.1), and 120 (6.7) mg/dL (mmol/L), and an automatic correction bolus delivery every 5 min. User-initiated meal announcements are required for optimal glycemic results. Every 5-min autocorrection improves daytime glycemia by mitigating inaccuracies in carbohydrate estimation and late or missed meal boluses, and accommodates for daily glucose variability without user intervention. Conditions for closed-loop exits were changed from those used in the MiniMed 670G algorithm to decrease user burden, while ensuring safety. A pivotal study assessing the performance and safety of the AHCL system was conducted in adolescents and adults, and demonstrated a mean TIR and HbA1c of 74.5% ± 6.9% and 7.0% ± 0.5%, respectively.^11^ When using the optimal system settings of a 100 mg/dL glucose target and active insulin time (AIT) of 2 h, users achieved an increased TIR of 78.8% ± 5.5%. In a separate AHCL system randomized controlled trial in children, adolescents, and adults,^12^ the proportion of users achieving TIR >70% increased from 12% at baseline to 51% during AHCL use.

The MiniMed 780G system received Conformité Europëenne (CE) Mark in June 2020, and is indicated for people with T1DM aged 7–80 years old, whose total daily dose of insulin is 8 U/day or more. Herein, we provide RWE on the performance of the MiniMed 780G system in 4120 users.

## Materials and Methods

MiniMed 780G system data uploaded to CareLink™ personal software from August 27, 2020, to March 3, 2021, by individuals who provided consent for their data to be aggregated were analyzed. IRB approval was waivered. Data from countries where the MiniMed 780G system was first introduced and from which local data privacy regulation permitted analyses were included in this analysis. These were Belgium, Finland, Italy, the Netherlands, Qatar, South Africa, Sweden, Switzerland, and the United Kingdom. Data are shown for specific countries that had ≥50 users. Users with ≥10 days of sensor glucose (SG) data after AHCL was enabled for the first time (post-AHCL) were included in the analysis, based on previous publications using a similar duration of time to estimate or determine CGM-derived metrics.^13,14^ All data available were included, whether the system was in AHCL control or in open loop (i.e., after an AHCL exit triggered by either the system or the user). Only users with >10 days of SG were included in the analysis for the GMI because the formula to compute GMI requires at least 10 days of CGM data.

Glycemic outcomes including the mean percentage of time spent between 70 and 180 mg/dL (3.9–10.0 mmol/L), <54 mg/dL (TBR <54) (<3.0 mmol/L), <70 mg/dL (TBR <70) (<3.9 mmol/L), >180 mg/dL (time above range, TAR >180) (>10.0 mmol/L), and >250 mg/dL (TAR >250) (>13.9 mmol/L) were determined for the overall 24-h day, daytime (06:01–11:59 h) and nighttime (12:00–06:00 h) periods. The mean SG levels and GMI were also assessed, as well as the sensor use, percentage of time spent in AHCL, number of self-monitored blood glucose (SMBG) measurements, insulin delivery patterns, and the system settings (i.e., glucose target and AIT).

The impact of initiating AHCL was evaluated by comparing the outcomes before AHCL was enabled for the first time (pre-AHCL) and after AHCL was enabled for the first time (post-AHCL) for users having ≥10 days of SG data in both periods.

### Statistics

There were two main cohorts analyzed: (1) a post-AHCL cohort composed of all individuals with ≥10 days of SG data after initiating AHCL for the first time (*N* = 4120) and (2) a pre- and post-AHCL cohort composed of individuals with ≥10 days of SG data in both the before AHCL initiation and after AHCL periods (*N* = 812).

The post-AHCL cohort underwent descriptive analysis using mean and standard deviation for continuous variables and proportion (%) for categorical variable. The pre- and post-AHCL comparison of glycemic outcomes was performed using a paired *t*-test in cases wherein the normality assumption was met, or a Wilcoxon signed-rank test. A McNemar's test was used for comparison of paired proportions. All statistical analyses were performed using SAS^®^ (version 9.4) software (SAS Institute, Cary, NC, USA) and *P-*values <0.05 were considered statistically significant.

## Results

### Overall performance of the MiniMed 780G system

A total of 6710 users from the 9 countries in the scope of the analysis uploaded data into CareLink personal software within the observation period, of whom 5327 provided consent for their data to be aggregated. There were 4120 users with ≥10 days of SG data post-AHCL initiation who were included in the analysis. This represented an overall 222,073 days of SG data and a mean users' follow-up of 54 ± 32 days. Users achieved a mean GMI of 6.8% ± 0.3% (coefficient of variation [CV]: 4.4%; interquartile range [IQR]: 6.5%–7.0%), TIR of 76.2% ± 9.1%, TBR <70 of 2.5% ± 2.1%, TBR <54 of 0.5% ± 0.7%, and TAR >180 of 21.3% ± 9.4% and TAR >250 of 4.2% ± 3.9%. The median (IQR) TIR achieved was 76.7% (70.8%–82.5%).

**FIG. 1.**
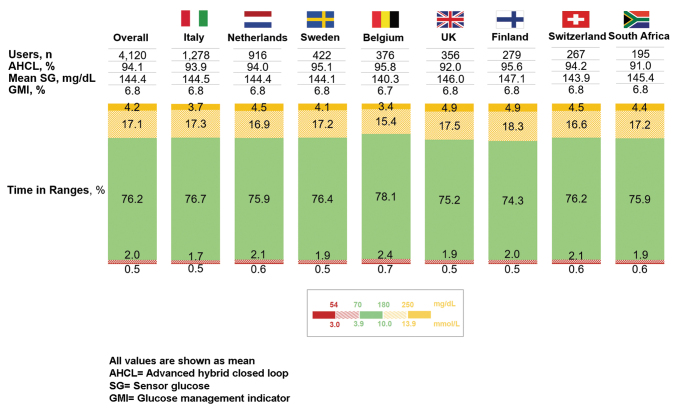
MiniMed™ 780G system performance after AHCL initiation, by country. AHCL, advanced hybrid closed loop.

There were 77.3% of users who achieved a >70% TIR and 79.0% who achieved a GMI of <7.0%. There were 80.9% and 83.3% of users who achieved a TBR <70 of <4.0% and TBR <54 of <1%, respectively, and 78.9% of users who achieved both TBR <70 and TBR <54 targets. Among the 8 countries with ≥50 users, the mean GMI ranged from 6.7% to 6.8%, TIR ranged from 74.3% to 78.1% and the TBR <70 ranged from 2.2% to 3.1% (Fig. 1).

Table 1 shows that the TIR during nighttime was significantly greater than that during the daytime (83.0% ± 11.2% and 73.9% ± 10.0%, respectively, *P* < 0.0001). The night and day TBRs <70 were 1.8% ± 2.4% and 2.8.0% ± 2.3%, respectively, and the night and day TBRs <54 were 0.4% ± 0.8% and 0.6% ± 0.8%, respectively (*P* < 0.0001 for both).

**Table 1.** Percentage of Time Spent Across Ranges Throughout the 24-H Day, Daytime and Nighttime Periods, Post-Advanced Hybrid Closed Loop Initiation

	Overall (24 h)	Day (06:01–11:59)	Night (12:00 − 06:00)	*P*
Time spent across ranges per day, %				
<54 mg/dL (3.0 mmol/L)	0.5 ± 0.7	0.6 ± 0.8	0.4 ± 0.8	<0.0001
<70 mg/dL (3.9 mmol/L)	2.5 ± 2.1	2.8 ± 2.3	1.8 ± 2.4	<0.0001
70–180 mg/dL (3.9–10.0 mmol/L)	76.2 ± 9.1	73.9 ± 10.0	83.0 ± 11.2	<0.0001
>180 mg/dL (10.0 mmol/L)	21.3 ± 9.4	23.4 ± 10.4	15.1 ± 11.3	<0.0001
>250 mg/dL (13.9 mmol/L)	4.2 ± 3.9	4.8 ± 4.5	2.5 ± 3.7	<0.0001

All values are shown as mean ± standard deviation; *P*-values indicate significant difference between day and night periods.

### System usability

The glucose sensor was used for a mean of 92.2% ± 8.3% of the time and the users were in AHCL for a mean of 94.1% ± 11.4% of the time. There was 0.9 ± 1.0 AHCL exits per week, including 0.4 ± 0.6 triggered by the system and 0.5 ± 0.9 triggered by the users (Table 2). Users performed an average of 3.4 ± 1.0 SMBG measurements per day.

**Table 2.** MiniMed™ 780G System Usability, Post-Advanced Hybrid Closed Loop Initiation

Sensor use, %	92.2 ± 8.3
AHCL, %	94.1 ± 11.4
SMBG measurements per day, *n*	3.4 ± 1.0
AHCL exits per week, *n*	
Total	0.9 ± 1.0
Initiated by the user	0.5 ± 0.9
Initiated by the system	0.4 ± 0.6
Glucose target level, % of time	
100 mg/dL (5.6 mmol/L)	50.3 ± 44.4
110 mg/dL (6.1 mmol/L)	22.2 ± 35.9
120 mg/dL (6.7 mmol/L)	21.6 ± 36.5
150 mg/dL (8.3 mmol/L)	1.7 ± 4.1
Manual mode	4.2 ± 8.8
AIT setting, % of time	
2 h	35.3 ± 45.9
>2–3 h	55.1 ± 47.2
>3–4 h	9.3 ± 27.2
>4 h	0.3 ± 4.7

All values are shown as mean ± standard deviation.

AHCL, advanced hybrid closed loop; AIT, active insulin time; SMBG, self-monitored blood glucose.

They selected the glucose target of 100 mg/dL (5.6 mmol/L) 50.3% of the time and an AIT of 2 h 35.3% of the time. Users selected an AIT of <3 h during 90% of the time (Table 2).

### Impact of initiating AHCL

**FIG. 2.**
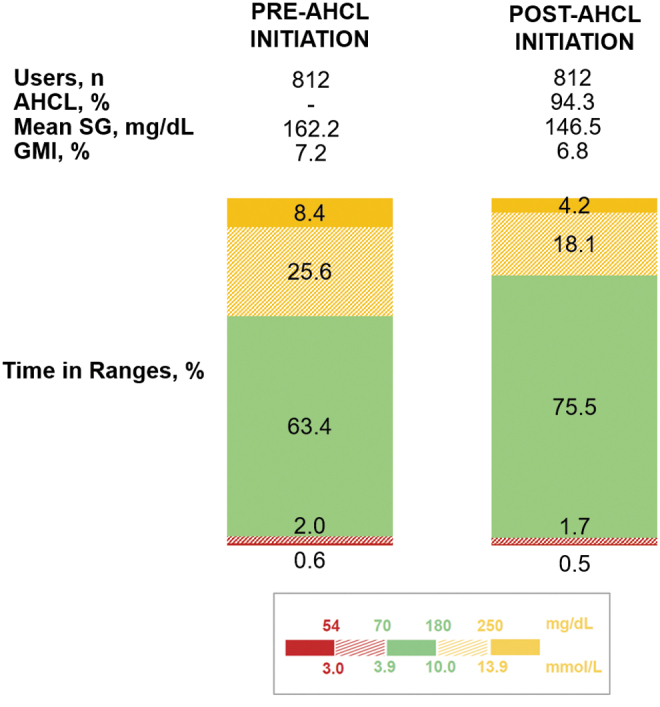
MiniMed™ 780G system performance pre- and post-AHCL initiation. Glycemic outcomes and time in AHCL are shown for MiniMed™ 780G system users with ≥10 days of SG data pre- and post-AHCL initiation. All differences between pre- and post-AHCL initiation values are statically significant (*P* < 0.0001 for all, except TBR <54 with *P* = 0.0005). TBR, time below range; SG, sensor glucose.

**FIG. 3.**
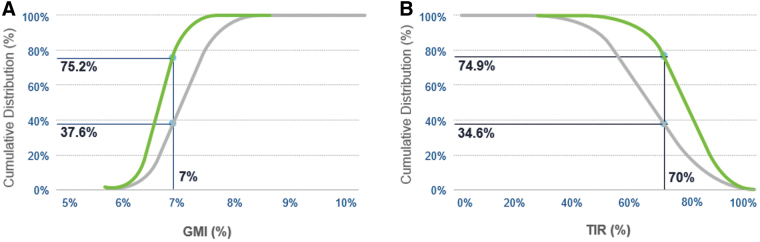
Percentage of users achieving recommended GMI and TIR goals with the MiniMed™ 780G system. Percentage of MiniMed™ 780G users achieving the international recommendations of <7.0% for the **(A)** GMI and >70% for the **(B)** percentage of time spent between 70 and 180 mg/dL (3.9 and 10.0 mmol/L), after initiating AHCL (black curve), compared with pre-AHCL initiation (gray curve). TIR, time-in-range; GMI, glucose management indicator.

There were 812 users who had ≥10 days of SG data both pre- and post-AHCL. Their mean SG improved significantly from 162.2 ± 23.1 mg/dL (9.0 ± 1.3 mmol/L) to 146.5 ± 14.9 mg/dL (18.1 ± 0.8 mmol/L) (*P* < 0.0001), after AHCL initiation (Fig. 2). Mean GMI decreased from 7.2% ± 0.5% to 6.8% ± 0.4% (*P* < 0.0001), TIR increased from 63.4% ± 14.3% to 75.5% ± 9.6% (*P* < 0.0001), TAR >180 decreased from 34.0% ± 15.3% to 22.3% ± 9.9% (*P* < 0.0001), and TAR >250 decreased from 8.4% ± 7.9% to 4.2% ± 4.1% (*P* < 0.0001). The TBR <70 decreased from 2.6% ± 2.6% to 2.2% ± 2.0% (*P* < 0.0001) and TBR <54 decreased from 0.6% ± 0.9% to 0.5% ± 0.6% (*P* = 0.0005). Figure 3 shows that more users achieved the glycemic treatment goals of GMI <7.0% (37.6% vs. 75.2%, *P* < 0.0001) and a TIR >70% (34.6% vs. 74.9%, *P* < 0.0001), after initiating AHCL, when compared with pre-AHCL initiation. The mean total daily dose (TDD) of insulin increased from 38.3 ± 18.7 U pre-AHCL initiation to 43.1 ± 22.0 U post-AHCL (*P* < 0.0001). This increase was mainly driven by auto corrections that accounted for 6.2 ± 4.8 U of insulin per day, representing 14.1% of TDD and 25.6% of total boluses. The user-initiated boluses decreased in both number and amount of insulin administered (Table 3). The number of SMBG measurements decreased from 4.7 ± 2.0 to 3.6 ± 1.2 from pre- to post-AHCL initiation (*P* < 0.0001).

**Table 3.** MiniMed 780G System Insulin Delivery, Pre- and Post-Advanced Hybrid Closed Loop Initiation

	Pre-AHCL initiation (*N* = 812)	Post-AHCL initiation (*N* = 812)	*P*
TDD of insulin, U	38.3 ± 18.7	43.1 ± 22.0	<0.0001
Basal, U (% of TDD)	18.0 ± 10 (46.9 ± 14.1)	—	0.028 (<0.0001)
Auto basal, U (% of TDD)	—	18.8 ± 10.3 (43.6 ± 8.5)	
Auto correction, U (% of TDD)	—	6.2 ± 4.8 (14.1 ± 6.0)	—
User-initiated bolus, U (% of TDD)	20.4 ± 11.7 (53.1 ± 14.1)	18.0 ± 10.1 (42.2 ± 11.0)	<0.0001 (<0.0001)
User-initiated boluses per day, *n*	6.0 ± 2.5	4.8 ± 2.0	<0.0001

All values are shown as mean ± standard deviation.

TDD, total daily dose.

## Discussion

RWE of a new therapy provides insights into whether or not results from relatively small and highly structured clinical trials can be generalized.^15^ Although randomized controlled trials provide a standardized mean by which to attribute effect of a therapy, real-world data provide a more realistic scenario to assess the outcomes of therapeutic interventions. Discrepancies between clinical studies and the real-world results are expected and have been reported as up to 27%.^16,17^ Clinical studies on insulin therapy usually exclude subjects prone to hypoglycemia or diabetic ketoacidosis (DKA), thus potentially biasing the estimation of adverse events in these trials. Indeed, higher rates of hypoglycemia are observed in real-world settings compared with clinical trial settings.^18^ Although episodes of severe hypoglycemia (i.e., requiring assistance) and DKA were not captured in our real-world analysis, it is reassuring that data from the 4120 users provide similar time spent at low glucose levels and within the target range as observed in the pivotal study.^11^

The reported glycemic outcomes achieved in this real-world analysis by users of the MiniMed 780G system were well within the recommended goals for all metrics as defined by the ADA Standards of Care for 2021,^19^ demonstrating a 76.2% TIR (recommended >70%), 2.5% TBR <70 (recommended <4%), 0.5% TBR <54 (recommended <1%), and TAR >180 and TAR >250 of 21.3% and 4.2% (recommended <25% and <5%, respectively). The mean GMI of 6.8% was also below the recommended goal of <7.0%. When assessing the percentage of users achieving the aforementioned goals, there were 79.0% who achieved the recommended goal for GMI <7.0%, and 77.3% and 78.9% who achieved the goals for TIR and time below ranges (both TBR <70 and TBR <54), respectively.

The extent of improvement in GMI observed in this study contrasts with the ∼20% of adults who achieved an HbA1c of <7% in the United Kingdom National Diabetes Audit^9^ and in the United States T1D Exchange,^20^ and 26.2% and 27.0% of participants in the western Europe and eastern Europe regions, respectively, of the SAGE study.^8^ Recent clinical studies addressing treatment with the automated insulin delivery system in the adolescent and young adult population have shown varied findings. A relatively lower rate of mean targets achieved, as well as the proportion attaining goals, has been reported in two RCTs of 3–6 months duration.^21,22^ However, a shorter RCT investigating the same AHCL system used in the FLAIR trial demonstrated greatest improvement in the TIR (+14.4%) for adolescents.^12^ Whether these results stem from differences in algorithms,^22^ suboptimal settings and reduced meal bolusing,^21^ or more optimized settings in a well-controlled group,^12^ the specific challenges in managing diabetes in youth will be assessed in future cohorts. Although the 6 months International Diabetes Closed-Loop (iDCL) randomized controlled trial in 168 participants (aged 14–71 years) with T1DM using a different AHCL system demonstrated a TIR that increased from 61% ± 17% at baseline to 71% ± 12%, in addition to significant improvements in TIR, TBR <70, TBR <54, and TAR >180 versus control,^23^ the proportion of individuals meeting consensus targets of HbA1c <7% was reported to be 47%.^22^ Two recent analyses of the same iDCL trial system in real-world use reported an overall group median TIR that increased from 63.6% to 73.6% after 1 year in >9000 individuals^24^ and to a mean of 71% in 700 users followed for 3 months.^25^

A characteristic of the MiniMed 780G system is its ability to achieve low variability in glycemic control by different users. Two lines of evidence support this: one is the low CV (4.4%) and narrow interquartile range (6.5%–7.0%) for the GMI achieved; the second is the low and clinically insignificant differences (<5% TIR and <0.2% GMI) achieved in different countries that differ in access, reimbursement, indications, as well as health care structure. This may be due to the robustness of the algorithm that provides treatment based on real-time glucose dynamics that can be personalized to the individual user's characteristics such as TDD of insulin and insulin effectiveness parameters that are adapted daily. This phenomenon was also demonstrated by the RWE of 14,899 users of the MiniMed 670G system,^26^ providing mounting evidence on the performance of AID therapies. The current cohort of MiniMed 780G system users showed better outcomes than those reported for real-world MiniMed 670G system users. We speculate that the incorporation of the auto correction bolus every 5 min, along with lower glycemic targets, was instrumental in providing improved overall glycemic control. Specifically, the auto correction may mitigate the effects of inaccurate meal carbohydrate assessments, inconsistent lifestyle or activity, and missed or late meal boluses.

The implications of these findings on the therapy for both users and providers are multiple. First, the system lowers the demands and burden on diabetes technology users compared with the MiniMed 670G system,^9^ as demonstrated by the high percentage of time spent in AHCL (94.1%), the low number of closed-loop exits (0.9/week), and the lower number of SMBG measurements performed after initiating AHCL. A reduction in user-initiated interactions was also observed, as improved glycemic control post-AHCL initiation was associated with significantly fewer user-initiated boluses (from 6.0 ± 2.5 to 4.8 ± 2.0) and lower amount of insulin delivered (from 20.4 ± 11.7 to 18.0 ± 10.1 U). The data also provide information on the system settings use. In the current real-world data analysis post-AHCL initiation, target glucose was set higher than the recommended 100 mg/dL for 50% of the time. During 10% of the time, AIT was set at >3 h while the system is optimized for an AIT of 2–3 h, suggesting that further optimization of settings may have provided even better glycemic outcomes. This is in line with a recently reported cohort of 52 individuals using the MiniMed 780G system with optimal settings of 100 mg/dL target and 2 h AIT, achieving a mean TIR of 79.6% ± 7.9% 1 month after AHCL initiation.^27^

The limitation of this analysis includes the lack of demographic data, such as users' duration of diabetes and previous therapies, as well as the number of users who may have discontinued and the associated cause, which are missing due to the anonymization required by the data privacy regulations. The usability can only be inferred from the high percentage of time spent in AHCL and the low number of AHCL exits. We are also limited by the follow-up duration of the cohort with a mean of 54 ± 32 days, although previous studies on the MiniMed 670G^26,28^ and MiniMed 780G^29^ systems demonstrated clinical benefits within the first weeks that are sustained over longer periods of up to 1 year. Further analyses are needed to evaluate the impact of the system over time and to identify which system settings allow for best outcomes.

The strength of this analysis is that it included a large number of users (*N* = 4120) contributing 222,073 days of SG data uploaded to the CareLink platform, providing assurance for the completeness of the data collected and mitigating selection bias. Our present cohort is only limited by the data privacy regulation and the requirement to assess users with a minimum of 10 days of available SG data.

In conclusion, the use of the MiniMed 780G system in 4120 people with T1DM in the real-world setting provided robust data on achievable glycemic control, while maintaining safety from hypoglycemia. When considering the current state of glycemic control, it is apparent that the promise of near euglycemia is achievable with AID therapies. This provides a compelling case for increasing access to these systems to people with T1DM.
